# Population Genomics of American Mink Using Whole Genome Sequencing Data

**DOI:** 10.3390/genes12020258

**Published:** 2021-02-11

**Authors:** Karim Karimi, Duy Ngoc Do, Mehdi Sargolzaei, Younes Miar

**Affiliations:** 1 Department of Animal Science and Aquaculture, Dalhousie University, Truro, NS B2N 5E3, Canada; karim.karimi@dal.ca (K.K.); duy.do@dal.ca (D.N.D.); 2 Department of Pathobiology, University of Guelph, Guelph, ON N1G 2W1, Canada; msargol@uoguelph.ca; 3 Select Sires Inc., Plain City, OH 43064, USA

**Keywords:** American mink, linkage disequilibrium, population structure, whole genome sequencing

## Abstract

Characterizing the genetic structure and population history can facilitate the development of genomic breeding strategies for the American mink. In this study, we used the whole genome sequences of 100 mink from the Canadian Centre for Fur Animal Research (CCFAR) at the Dalhousie Faculty of Agriculture (Truro, NS, Canada) and Millbank Fur Farm (Rockwood, ON, Canada) to investigate their population structure, genetic diversity and linkage disequilibrium (LD) patterns. Analysis of molecular variance (AMOVA) indicated that the variation among color-types was significant (*p* < 0.001) and accounted for 18% of the total variation. The admixture analysis revealed that assuming three ancestral populations (K = 3) provided the lowest cross-validation error (0.49). The effective population size (*Ne*) at five generations ago was estimated to be 99 and 50 for CCFAR and Millbank Fur Farm, respectively. The LD patterns revealed that the average *r*^2^ reduced to <0.2 at genomic distances of >20 kb and >100 kb in CCFAR and Millbank Fur Farm suggesting that the density of 120,000 and 24,000 single nucleotide polymorphisms (SNP) would provide the adequate accuracy of genomic evaluation in these populations, respectively. These results indicated that accounting for admixture is critical for designing the SNP panels for genotype-phenotype association studies of American mink.

## 1. Introduction

Characterizing the genetic structure is crucial to reveal the genetic diversity, domestication history, and genetic relationship of populations that eventually facilitate the development of efficient breeding strategies in domestic animals [1]. Advances in DNA sequencing technologies have provided the opportunity to use a large number of single nucleotide polymorphisms (SNPs) for investigation of genetic structure and diversity of livestock species, e.g., cattle [2,3], sheep [4], goat [5], and pig [6]. In addition, these markers are efficient tools to measure the linkage disequilibrium (LD) across the genome [7]. The LD refers to the non-random association of alleles at two or more loci and is indicative of the correlation between two nearby loci [8]. The LD might be caused by physical proximity between markers located on the same chromosome (linkage) whereas it is eroded by recombination events [9]. In addition, LD could be generated between unlinked markers due to several factors including genetic drift, selection, epistatic combinations and population structure [7]. For instance, admixture between distinct populations can arise LD between two unlinked loci, particularly those with different allele frequencies [10,11].

The LD levels are used as a measure to determine the required density of markers for genome-wide mapping studies [12]. Moreover, the magnitude and decay of LD over genomic intervals can be used to estimate the recent and past effective population sizes (*Ne*). Since recombination takes less time to break down the LD between loosely linked loci, LD at longer distances reflects *Ne* at recent generations whereas LD from physically linked loci is an indicator of *Ne* at past generations [13,14]. The *Ne* is the key parameter to monitor the evolutionary history, inbreeding risk and conservation priority in animal populations [15,16,17]. Furthermore, assessment of relationship among individuals using molecular markers provides more accurate measures of inbreeding levels to manage ongoing mating [18].

American mink (*Neovison vison*) is a semiaquatic species of Mustelid family, which is known as one of the most popular resources of fur worldwide [19]. There is no much information available on the domestication history in American mink. However, it was stated that the American mink was initially bred in captivity in 1866 to produce fur [20]. Whereas some farmers tend to raise a particular color-type of mink, most farms are mixed of different color-types. Market demands may affect the color combination of mink farms. Unfortunately, most farms have no regular mating system and animals are mostly selected based on the phenotypic performances meaning that incidental gene flow could happen among populations [21,22]. Pelt size, fur quality, reproductive performance, and disease resistance are the main traits of interest for mink breeders [23,24,25]. Implementation of highly efficient breeding strategies is critical for the mink industry to adopt sustainable production practices, e.g., increasing resiliency against emerging diseases, resolving the ethical issues of fur production and reducing the cost of production [25]. Despite the successful utilization of genomic selection approaches in improvement of genetic merits in other livestock species, e.g., dairy cattle [26] and pig [27], this breeding strategy has not been applied in the mink industry. Whole genome sequence data provide a large number of SNP makers that can be used for genome-wide studies in American mink and accelerate the development of modern genomic selection in this industry [28,29].

Analysis of genetic structure and genome-wide LD pattern of mink populations can provide fundamental information for developing tools required for genomic selection in American mink. The genetic structure of farm and feral American mink was previously studied using 11 microsatellite markers in Japanese mink [30], 154 SNPs generated from restriction-site associated DNA sequencing (RAD-seq) in Polish and Danish mink populations [31], and mitochondrial DNA sequences along with 11 microsatellite markers in Chilean mink [32]. Microsatellite DNA markers were also used to estimate the *Ne* in American mink based on the LD information [33,34]. Moreover, 13,321 SNPs extracted by genotyping-by-sequencing (GBS) was used to investigate the LD patterns and estimate the *Ne* of black American mink [35]. However, there is no study on the LD patterns and *Ne* of different color-types of American mink using the whole genome sequencing data. In addition, admixture patterns raised by the presence of different color-types on mink farms would affect the extent of LD, which is not well-characterized in American mink. Therefore, the main ideas of this study were (1) to determine the genetic structure and admixture pattern of various color-types of American mink in Canada, (2) to investigate the LD pattern across the genome for estimation of the required marker density for genome-wide studies in American mink, and (3) to estimate the genomic inbreeding coefficients and *Ne* in two mink farms in Canada.

## 2. Materials and Methods

### 2.1. Animals and Sampling

All protocols were approved by the Dalhousie University Animal Care and Use Committee (certification number: 2018–009), and mink used in this study were cared for according to the Code of Practice for the Care and Handling of Farmed Mink guidelines [36]. Mink were housed under standard farming conditions and humanly euthanized in December 2018 [24]. Tongue samples of 100 individuals were collected from two farms including the Canadian Center for Fur Animal Research (CCFAR) at Dalhousie Faculty of Agriculture (Truro, NS, Canada) and Millbank Fur Farm (Rockwood, ON, Canada). Animals from CCFAR consisted of various color-types including Demi (*n* = 32), Pastel (*n* = 10), Mahogany (*n* = 20), Stardust (*n* = 7) and Black (Black-NS, *n* = 16). All animals from Millbank Fur Farm (*n* = 15) were of Black color-type (Black-ON). The relationship between animals was checked based on the pedigree information and only animals with less degree of kinship were selected for further analyses (the median = 0.015; 1st–3rd quantile of relatedness = 0.008–0.039).

### 2.2. Whole Genome Sequencing and Variant Calling

Genomic DNA was extracted from tongue tissue using DNeasy Blood and Tissue Kit (Qiagen, Hilden, Germany) following the manufacture protocol. DNA samples were then sequenced (100 bp pair-end reads) using the BGISEQ-500 platform at Beijing Genomics Institute (BGI, Guangdong, China). After sequencing, SOAPnuke software [37] was used to filter out sequencing adapters and low-quality reads. The clean reads were mapped to the American mink reference genome [38] using the mem-algorithm of the Burrows Wheeler Aligner (BWA) software [39]. The aligned files were converted from sequence alignment map (SAM) to binary alignment map (BAM) format and sorted using SAMtools package version 1.11 [40]. Potential PCR duplications were removed using ‘MarkDuplicates’ of Picard [41]. The BAM files were then indexed by SAMtools. Finally, variant calling was performed with the ‘mpileup’ module of SAMtools and BCFtools. All variants were filtered using Variant Call Format (VCF) tools based on the following parameters: minor allele frequency (MAF) >0.05; maximum missing rate <0.1; minor quality >30; and only bi-allelic variants were kept.

### 2.3. Inbreeding Coefficients and Genetic Distances

The average MAF, heterozygosity levels and inbreeding rate based on excess of homozygosity (F_HOM_) were estimated for each color-type using SNP1101 version 1.0 [42]. Runs of homozygosity (ROH) were detected for each individual using SNP1101 software. The following parameters were set to detect ROH: minimum window size of 20 SNPs, genotyping error rate of 0.01 and sliding window step size of one SNP. The minimum length of ROH was set to be 500 kb, 1 Mb and 2 Mb in different runs. The inbreeding coefficients based on ROH (F_ROH_) were calculated using Equation (1) as follows [43]:

(1) $$F_{\mathrm{ROH}}=\frac{\sum_{K}\left(\mathrm{Length}\left({\mathrm{ROH}}_{K}\right)\right)}{L},$$

where $\sum_{K}\left(\mathrm{Length}\left({\mathrm{ROH}}_{K}\right)\right)$ indicates the total length of ROHs above a threshold length and L is the total length of genomic regions in which SNPs could be called, which was 802 Mb in this study.

Pairwise genetic distances were computed according to Nei [44] and Weir and Cockerham [45] procedures using ‘StAMPP’ package of R [46]. In addition, analysis of molecular variance (AMOVA) was performed using StAMPP package to describe the genetic variation among samples at different hierarchical levels. The AMOVA was computed based on the Nei’s genetic distance matrix using the stamppAmova() function following the procedure described by Excoffier et al. [47].

### 2.4. Genetic Structure and Admixture Patterns

Discriminant analysis of principal components (DAPC) was used to infer the genetic structure of the studied color-types. The DAPC was performed based on the procedure implemented in the ‘adegenet’ package of R [48]. Adegenet implements a prior principal component analysis (PCA) to transform the data and subsequently performs the discriminant analysis. The pre-defined number of clusters was determined using k-means algorithm, which found a given number (K) of clusters maximizing the variation between the clusters. The number of clusters between one and ten was explored and the Bayesian Information Criterion (BIC) was used to choose the optimum number of clusters following the elbow method. The number of retained principal components (PCs) was selected by the a-score [48,49].

Population structure and potential admixture among the populations (color-types) was further assessed with an unsupervised analysis in ADMIXTURE version 1.3.0 [50]. The ADMIXTURE was run for K = 1 to K = 6 with ten iterations per K. A cross-validation procedure was applied to infer the best number of ancestral populations (K). The K value with the lowest standard error of cross-validation error was assumed as the best indicator of admixture pattern among populations.

### 2.5. Linkage Disequilibrium (LD) and Effective Population Size

The LD analysis and *Ne* estimation were performed based on the data of 100,000 SNPs randomly selected across scaffolds >10 Mb. The *r*^2^ statistic was computed to measure the LD level between all possible pairs of SNPs located on the same scaffold using SNP1101 software. Equation (2) was used to estimate the *r*^2^ values [51]:

(2) $$r^{2}\;=\;\frac{D^{2}}{\left(P_{A}P_{B}P_{a}P_{b}\right)},$$

where: *D* = P_AB_−P_A_P_B_ and P_A_, P_a_, P_B_, and P_b_ are the frequencies of alleles A, a, B, and b, respectively; and P_AB_ presents the frequency of haplotype AB. Since haplotypes were not reconstructed, an unbiased estimator of D was computed using Equation (3) [52]:

(3) $$D=\;\frac{N}{N-1}\;\left[\frac{4N_{\mathrm{AABB}}+2\left(N_{\mathrm{AABb}}+N_{\mathrm{AaBB}}\right)+{\;N}_{\mathrm{AaBb}}}{2N}-P_{A}P_{B}\right],$$

where: N is the total number of samples and N_AABB_, N_AABb_, N_AaBB_, and N_AaBb_ are the number of genotypes AABB, AABb, AaBb and AaBb, respectively. In addition, sample size correction for unphased data was made to all computed *r*^2^ using Equation (4) as [53,54]:

(4) $$r_{corrected}^{2}=\;\frac{r_{computed}^{2}-\;\frac{1}{n}}{1-\;\frac{1}{n}},$$

where *n* is the number of sampled haplotypes. The LD decay was assessed in three distance sets of ≤100 kb, ≤1000 kb, and ≤10 Mb, and SNP pairs were binned in each distance set using bin sizes of 10 kb, 100 kb, and 1000 kb, respectively. The average *r*^2^ of each bin was plotted against the median size of the bin to depict the LD decay across the genome distances.

In addition, Equation (5) was used to estimate the effective population size based on the expectations for r^2^ over different genomic intervals [55]:

(5) $$r^{2}\;=\;\frac{D^{2}}{\left(P_{A}P_{B}P_{a}P_{b}\right)},$$

where: c shows the median of the recombination distance between SNPs in Morgans. The age of Ne at a given distance was estimated by 1/(2c) as suggested by Hayes et al. [56]. The recombination rate was assumed to be 1 centiMorgan per million base pairs. The *Ne* was assessed at 200, 150, 100, 50, 10, and 5 generations ago to monitor the changes in population size.

## 3. Results

### 3.1. Data Quality and Population Genetic Parameters

In total, more than 100 billion reads were generated with a range of 817,199,893 to 1,050,757,227 reads per sample. The genome coverage was in the range of 34× to 44× with an average of 40× per sample. On average, 98.24% of reads were mapped to mink reference genome, with the range of 97.93 to 98.55%. In total, 22,990,329 variants were called by SAMtools and BCFtools and after quality control, 8,150,569 bi-allelic variants from 100 individuals were remained for further analysis.

Table 1 presents the average MAF, observed heterozygosity, F_HOM_, and pedigree based inbreeding rate (F_PED_) in different color-types. The average MAF ranged from 0.190 (Stardust) to 0.214 (Demi). The lowest level of heterozygosity (28.03%) was observed in Black-ON whereas the Black-NS had the highest percentage of heterozygosity (31.32%). The F_HOM_ ranged from −0.189 in Demi to −0.112 in Black-ON. The highest average of F_PED_ was found in Pastel (0.026) and Demi had the lowest value of F_PED_ (0.006). The correlation between F_HOM_ and F_PED_ was estimated to be 0.59.

Table 2 shows the average (±SD) F_ROH_ and the number of ROH segments detected in different studied groups based on the minimum ROH lengths of 500 kb, 1 Mb and 2 Mb. The highest average number of homozygous segments (120 ± 21) and F_ROH_ (0.138 ± 0.031) based on the minimum length of 500 kb were observed in Black-ON. The lowest average of F_ROH_ > 500 kb (0.072 ± 0.022) was found in Demi with an average (±SD) of 61 ± 17 segments per individual. Similar trends were also observed for higher thresholds of ROH length (Table 2). The short segments (500 kb to 1 Mb) included the most proportion of ROH detected among samples (69%). The highest number of identified ROH (length> 500 kb) per individual was 180, which was detected in one Black-ON mink. The correlations of F_ROH_ (length >500 kb) with F_HOM_ and F_PED_ were 0.85 and 0.47, respectively.

### 3.2. Genetic Distances

Table 3 presents the Weir and Cockerham’s Fst and Nei’s genetic distance among different color-types. The highest Fst (0.124) and Nei’s genetic distance (0.065) were found between Pastel and Stardust color-types. Both measures indicated that Demi and Mahogany color-types had the minimum genetic distances.

Table 4 shows the AMOVA for the studied color-types. The higher proportion of variation was explained by the differentiation within color-types (73%). The variation among color-types was also significant (*p* < 0.001) and represented 18% of the total molecular variation in the populations. On the other hand, the variation among farms was not significant and accounted for 9% of the total variation.

### 3.3. Genetic Structure and Admixture Patterns

**Figure 1:**
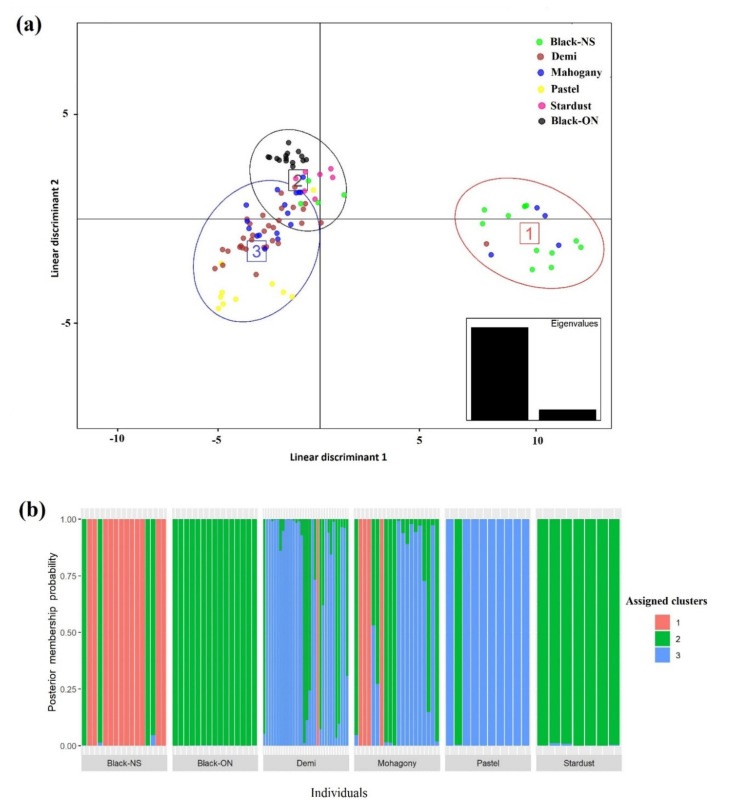
Discriminant analysis of principal components (DAPC) of five color-types of American mink: (**a**) The scatter plot of the first two linear discriminants for all samples. Color-types are presented by different colors and the three main clusters are shown by circles and numbers. The first two linear discriminants explained 89.68 and 10.32% of the variation, respectively; (**b**) The probability of membership of each sample in the three assigned clusters inferred by DAPC. Black-NS and Black-ON represent the black color-type samples collected from the Canadian Center for Fur Animal Research (Truro, NS, Canada) and Millbank Fur Farm (Rockwood, ON, Canada), respectively.

The lowest value of BIC was observed for *K* = 3 indicating the most likely number of genetic clusters in the data set. The DAPC was performed to reveal the population structure assuming three main clusters. Figure 1a presents the scatter plot of the first two linear discriminants (LD) for all samples. Most of Black-NS individuals were placed in a separate cluster. Whereas Mahogany and Demi shared the overlapping clusters, Pastel, Stardust and Black-ON were assigned to certain clusters. In addition, the membership probabilities of each individual to different clusters were presented in Figure 1b. The Black-ON and Stardust shared the same cluster and most Pastel and Black-NS were assigned to other two unique clusters. However, Demi and Mahogany were largely admixed from other clusters.

**Figure 2:**
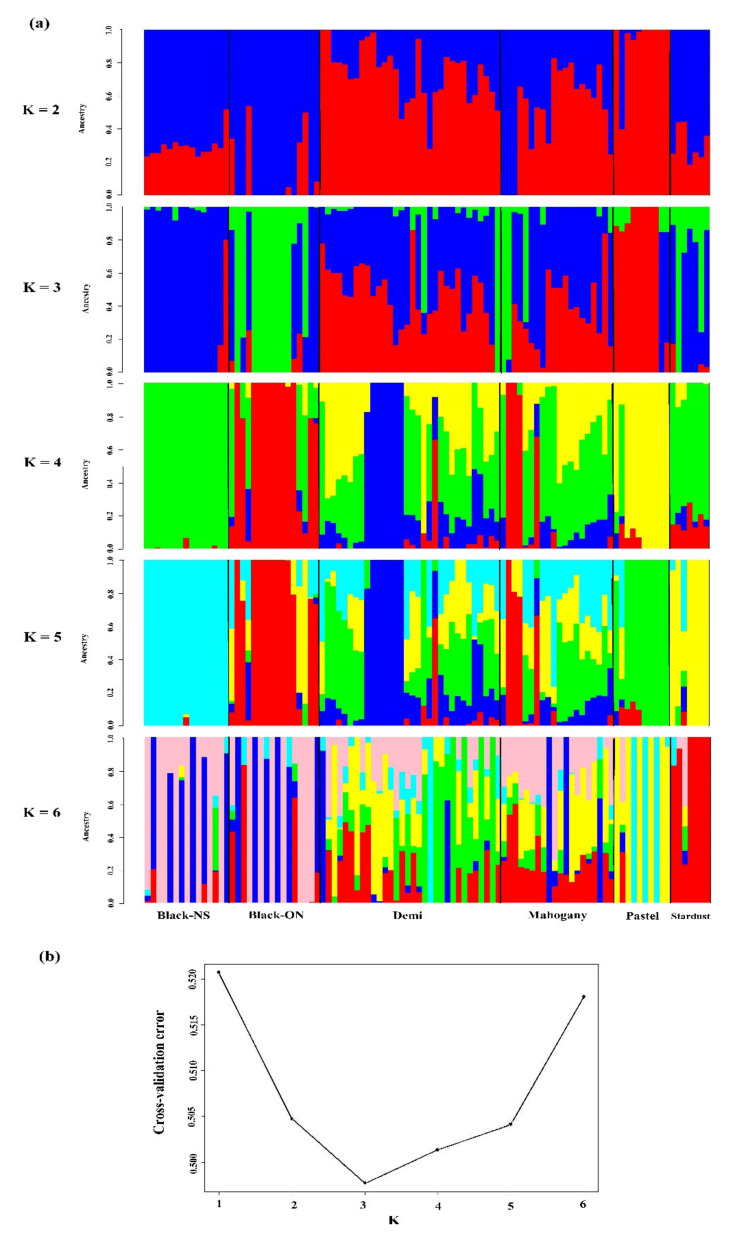
Admixture patterns of American mink color-types: (**a**) Admixture analysis at *K* = 2 to *K* = 6 in five color-types of American mink. Each individual was represented by a vertical bar and different colors were used to present color-types; (**b**) Cross-validation error as a function of K for admixture analysis of five color-types. The lowest cross-validation error was found at *K* = 3. Black-NS and Black-ON represent the black color-type samples collected from the Canadian Center for Fur Animal Research (Truro, NS, Canada) and Millbank Fur Farm (Rockwood, ON, Canada), respectively.

We also used the admixture analysis to reveal the admixture patterns assuming two to six ancestral populations (Figure 2). The lowest cross-validation error (0.49) was found for *K* = 3, indicating the best fitting model of admixture among the studied individuals (Figure 2b). Ancestral proportions at *K* = 3 indicated that Black-ON individuals were mainly assigned to the same cluster (on average, 92.36%). In addition, Pastel (on average, 78.17%) and Black-NS (on average, 62.94%) constructed two other main genetic compositions whereas other color-types (Demi, Mahogany, and Stardust) were admixed of these three clusters (Figure 2).

### 3.4. Linkage Disequilibrium and Effective Population Size

**Figure 3:**
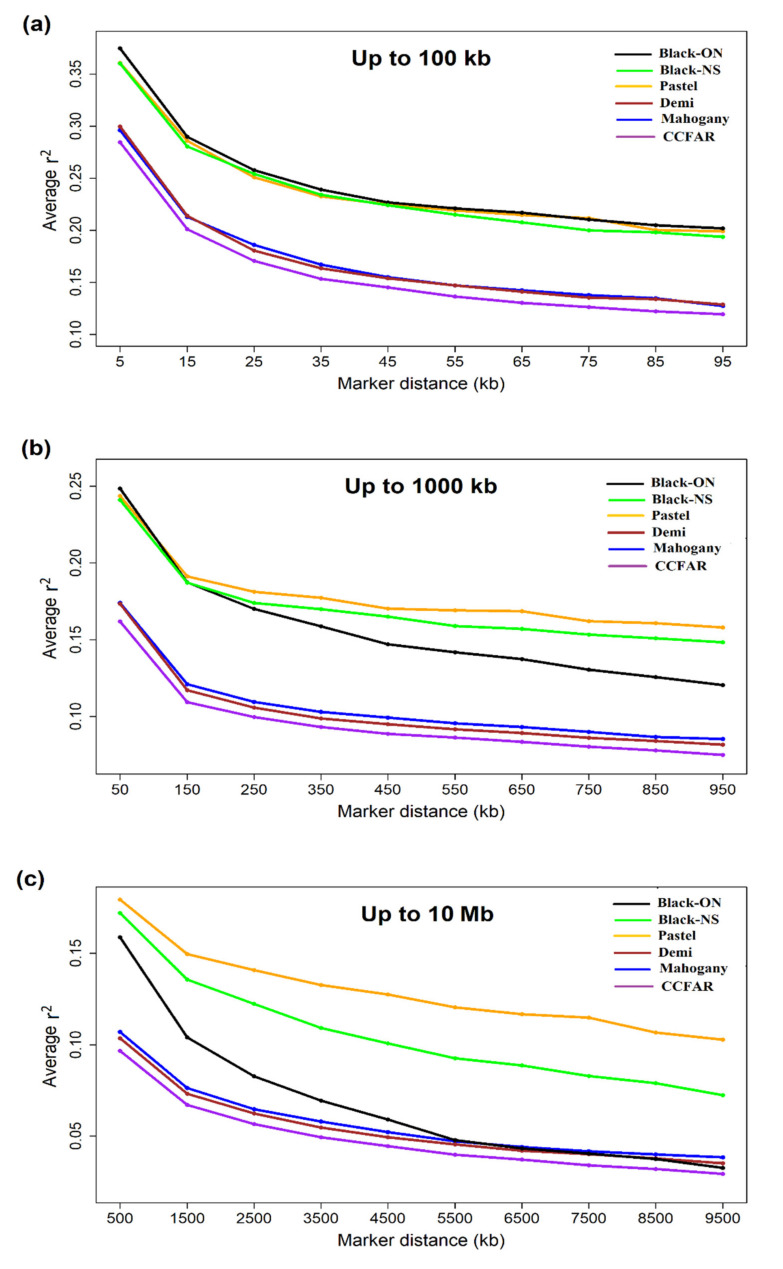
Linkage disequilibrium (LD) decays represented by the average r^2^ for inter-marker distances of (**a**) up to 100 kb using consecutive 10 kb bins (**b**) up to 1000 kb using consecutive 100 kb bins and (**c**) up to 10 Mb using consecutive 1000 kb bins. Black-NS and Black-ON represent the black color-type samples collected from the Canadian Center for Fur Animal Research (Truro, NS, Canada) and Millbank Fur Farm (Rockwood, ON, Canada), respectively.

Table 5 shows the average *r*^2^ ± SD between adjacent SNPs for different color-types. In addition, the average *r*^2^ ± SD between adjacent SNPs over all studied scaffolds was presented in Table S1. The average distance between adjacent markers was 8.17 ± 2.84 kb. The highest and lowest average *r*^2^ ± SD between adjacent markers were observed in Black-ON (0.366 ± 0.388) and Mahogany (0.280 ± 0.361) color-types, respectively. We also computed the pairwise *r*^2^ between markers to describe the LD decay in different genomic distances. Figure 3 presents the LD decay up to 100 kb, 1000 kb, and 10 Mb using 10 kb, 100 kb, and 1 Mb bins, respectively. In addition, Table S2 presents the extension of LD (average *r*^2^ ± SD) at different intervals up to 1000 kb. The average *r*^2^ > 0.2 extended to intervals up to 20 kb in Demi and Mahogany. On the other hand, higher levels of LD (average *r*^2^ > 0.2) were observed for intervals up to 80 kb, 90 kb, and 100 kb in Black-NS, Pastel and Black-ON color-types, respectively. More rapid reduction was observed in LD decay of Black-ON over 200 kb genomic intervals (Figure 3).

**Figure 4:**
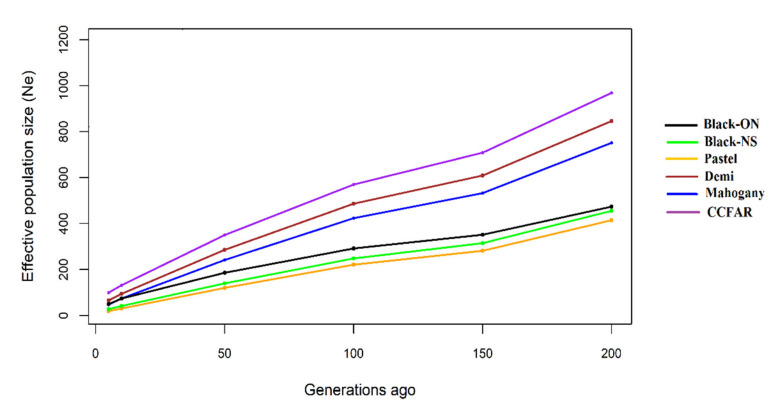
Estimated effective population sizes (*Ne*) for five color-types of American mink as a function of generations ago. Black-NS and Black-ON represent the black color-type samples collected from the Canadian Center for Fur Animal Research (Truro, NS, Canada) and Millbank Fur Farm (Rockwood, ON, Canada), respectively.

The *Ne* was also estimated based on the average LD obtained in different genomic intervals. Figure 4 represents the changes of estimated *Ne* over the last 200 generations. The recent *Ne* (five generations ago) was estimated to be 19, 47, 66, 28, and 50 in Pastel, Mahogany, Demi, Black-NS, and Black-ON, respectively. The recent *Ne* was 99 for all individuals from CCFAR farm. Furthermore, the maximum *Ne* was observed for Demi (846) at 200 generations ago whereas the lowest population size was for Pastel (414) at the same time.

## 4. Discussion

This study used the whole genome sequence data to assess the genetic structure of 100 American mink from two Canadian mink farms. The results of this study confirmed that there is no wide genetic differentiation among studied color-types owing to high levels of gene flow. However, these findings were critical to determine the markers density required for genomic studies in the mink farms.

High percentage of reads mapped to the reference genome (on average, 98.24%) confirming the high efficiency of the whole genome sequence data in this study. The number of SNPs obtained in this study (8,150,569) was much higher than 52,714 and 34,816 SNPs that were extracted by GBS technique to study the LD pattern [35] and the genes associated with body size [57] in American mink, respectively. This is due to the fact that GBS is a restriction enzyme-based technique that can only sequence a reduced subset of genome [58]. These results are promising to design the SNP panel using whole genome data and subsequently develop the genome-enabled selection in American mink.

The F_HOM_ ranged between −0.189 and −0.112 in different color-types (Table 1), that were less than the average value of 0.150 obtained from 52,714 SNPs in black American mink [35]. This discrepancy can be due to the differences in the historical background, sampling approaches and the density of markers applied in these studies. In addition, the higher genomic inbreeding rate of 0.271 was reported using microsatellite markers for farm and wild American mink in Eastern Canada [59], which was attributed to line-breeding in the studied farms, and breeding between related individuals, as well as a limited movement of wild mink. On the other hand, inbreeding coefficients were in the range of −0.150 to 0.005 using a panel of 194 SNPs for farm and feral American mink in Denmark and Poland, which were in agreement with those (−0.189 to −0.112) observed in the current study. The lowest level of heterozygosity (28.03%) was observed for Black-ON, which was in agreement with the higher degree of inbreeding (−0.112) estimated for this group. The highest level of inbreeding based on pedigree was found for Pastel (0.026) that was in accordance with the low *Ne* (19) estimated for this color-type. The high correlation of 0.59 was observed between F_PED_ and F_HOM_ across different color-types. This value is comparable with the correlations of 0.58, 0.48, and 0.51 reported in three cattle breeds [60], seven pig breeds [61] and Spanish Holstein population [62], respectively.

The inbreeding rates were also investigated using ROH analysis. The highest average F_ROH_ was observed for Black-ON across all the defined lengths, which was in accordance with the higher F_HOM_ (−0.112), lower heterozygosity (28.03%) and *Ne* (50) found in this group. The average F_ROH_ > 500 kb ranged from 0.072 to 0.138 among different groups in this study, which were lower than 0.186 reported for black American mink using 13,321 SNPs [35]. These differences might be attributed to the genetic history, sample size, coverage of genome and density of markers existed in these studies. The length of homozygous segments can provide evidence to infer the recent and ancient inbreeding events where the short autozygous tracts are related with ancient inbreeding and longer segments correlate with recent inbreeding events [63]. It was approximated that the ROHs >1 Mb date back about 50 generations ago [64]. The abundance of shorter ROH (500 kb to 1 Mb) indicated that the inbreeding events likely occurred over 50 generations ago in the studied populations, which falls into the approximated time of reduction in population sizes due to domestication [20]. Since there is no chromosome-scale genome assembly available for American mink [38], the analyses of this study were restricted to longer scaffolds (>10 Mb). Accordingly, it was not possible to assess the recent inbreeding events across the populations based on the detection of longer ROH. The availability of chromosome-scale genome assembly for American mink can open more opportunities to reveal the genetic diversity and develop the genomic studies of this species. High correlation of 0.85 was found between F_HOM_ and F_ROH_, which was comparable with the values of 0.87, 0.89, and 0.83–0.95 reported in black American mink [35], four cattle breeds [65] and Italian local cattle [66], respectively. However, a lower correlation (0.47) was observed between F_ROH_ and F_PED_. These results implied that genomic inbreeding coefficients can provide more accurate measure to control assortative mating on mink farms.

Although the AMOVA indicated that the genetic differentiation among color-types was significant, this explained a low proportion of total variance (18%) was explained by that. The multivariate DAPC could also differentiate color-types in certain groups; however, clusters were overlapped and the distances between groups were not considerable, which might be due to high gene flow and the admixture events occurred in recent generations among studied mink. The Black-ON samples were closely clustered in a separate group whereas the Black-NS individuals were mainly grouped in another cluster, showing the differences between two black color-types in this study (Figure 1). These results revealed that geographical distribution may play more important role in genetic differentiation of mink compared to color-types. It seems that extensive admixture events have shaped the genetic structure among mink color-types in CCFAR farm (Figure 1b and Figure 2), which was expected due to raising different color-types on the farm without systematic mating. However, Pastel and Black-NS were recognized as two main genetic compositions constructing the genetic structure of the studied individuals from CCFAR. Assuming three genetic clusters, Black-ON was the phenotypic category with the least evidence of admixture as all individuals were inferred to be largely from one ancestry component (Figure 1b and Figure 2a). The low genetic distances indicated that there was no extensive discrimination among the studied color-types (Table 3). Although the color-types existing in the CCFAR were not widely differentiated, the intensity of admixture and inbreeding rates were not similar across the color-types due to the differences in the group sizes, historical background and unmanaged mating in the past generations. In accordance with the result of this study, Thirstrup et al. [31] observed low genetic differentiation (Fst) among mink color-types (0.076) and mink from two different geographical origins (0.087). However, pairwise Fst ranged between 0.017 and 0.364 among mink from 12 locations in southern Chile [32], which were higher than those observed in this study (0.013 to 0.065). Besides the genetic origins and sampling methods, the differences in the marker types and density can be possible reasons for the higher Fst values in the Chilean mink populations.

The estimations of *Ne* at five generations ago were 99 and 50 for CCFAR and Millbank Fur Farm, respectively. These estimations were less than the *Ne* of 116 observed in black American mink using SNP data extracted by GBS technique [35]. This difference might be related to the history of populations, sample sizes, and density of markers used in these studies. In addition, the range of 17.5 to 78.8 [34] and 7.2 to 34.8 [33] were estimated using microsatellite markers for mink population size in Swedish coasts and Spain, respectively. The low *Ne* estimated in these studies were attributed to the conservation programs conducted in these areas to control the mink population size. Furthermore, the discrepancy of estimations might be due to the differences in the density and type of DNA markers, and genetic background of populations. Despite the different estimations for *Ne* among the studied color-types, the similar trends were observed for changes in *Ne* from 200 to 5 generations ago (Figure 4). Our results indicated that the decline of population sizes was more rapid between 200 and 150 generations ago, which was in accordance with the domestication events initiated to produce fur in 1866 [20]. These trends were in agreement with those reported for black American mink using GBS data [35]. It is suggested to use the high-throughput genomic data to control the reducing population size and levels of inbreeding in mink farms.

The average *r*^2^ between adjacent markers were in range of 0.280 to 0.366 for different color-types, which was in agreement with the value of 0.300 ± 0.350 reported by Karimi et al. [35] for American mink. Genomic selection and genome wide association studies (GWAS) rely on the assumption of the presence of LD between markers and the genes controlling the traits of interest [67]. The *r*^2^ of > 0.2 was suggested as the required LD level between casual gene and marker to achieve an accuracy of > 0.85 in genomic selection programs [68]. Similarly, *r*^2^ > 0.3 can be accounted as the threshold LD to obtain adequate power and precision in GWAS [7]. In this study, the LD patterns revealed that the average *r*^2^ reduced to <0.2 at inter-marker intervals of >20 kb and >100 kb in CCFAR and Millbank Fur Farm, indicating that the density of 120,000 (2.4 Gb/20 kb, where 2.4 Gb is the size of genome assembly) and 24,000 (2.4 Gb/100 kb) SNPs would provide the acceptable accuracy for genomic selection in these populations, respectively. Whereas the average *r*^2^ > 0.2 was extended to marker distances of <20 kb for both Demi and Mahogany, the higher extensions of 80 and 90 kb were observed for Black-NS and Pastel, respectively. In addition, the average *r*^2^ >0.3 at genomic distances of <10 kb for both CCFAR and Millbank Fur Farm suggested that 240,000 informative SNPs can capture the LD levels necessary for GWAS in these populations. Based on the GBS data, Karimi et al. [35] suggested the density of 60,000 SNPs for genomic selection in black American mink, which is higher than the 24,000 suggested for Millbank Fur Farm, but is less than the 120,000 for CCFAR in this study. In addition, the 120,000 SNPs suggested by Karimi et al. [35] for GWAS is less than the density of 240,000 required for both farms in the current study.

Our results indicated that higher marker densities are required for admixed population (CCFAR) compared to non-mixed population (i.e., Millbank Fur Farm). Similar to our results, lower LD was observed for a group of admixed Danish Jersey cattle in comparison with the original Danish and United States Jersey dairy cattle whereas the LD persistence was lower at longer marker distances for the original groups [69]. In addition, lower LD levels were reported at short distances for a heterogeneous breed (Rangeland) in Australian and Canadian goat breeds, suggesting that higher dense panel is required for genomic selection in this breed [70]. Admixture can generate the LD between both linked and unlinked loci, particularly those with different allele frequencies in the founder populations [11]. Although admixture-induced LD (ALD) between unlinked loci disappears quickly owing to recombination events, ALD between linked loci will persist for several generations. Despite the increasing levels of long-range ALD, the short-range LD, which is originated from the founder populations, is simultaneously reduced due to the admixture events [69,71]. Although sparse markers can effectively capture the long-range ALD, only a limited proportion of genetic variation is likely to be explained, restricting the accurate genomic predictions. The efficiency of genomic selection in the admixed population relies on the LD levels remained from founder populations, the long-range LD induced by admixture, and the degree of genetic differentiation between original populations [71]. The significance of these factors depends on the heritability, genetic architecture of the traits, density of markers, and the genomic selection approaches applied in the data [71,72]. In the mink farms, the traits of interest are usually measured in mixed populations with no certain genetic composition. This implies that not accounting for admixture pattern can bias the estimates of marker effects for genomic selection [72]. Our results confirmed that admixed population would require a higher density of SNPs for genomic selection and GWAS.

The results revealed that three main genetic compositions recognized by admixture analysis (i.e., Pastel, Black-NS and Black-ON) presented the higher levels of LD whereas the admixed color-types (Mahogany and Demi) had lower LD levels (Figure 3). The trend of LD decay and *Ne* suggested that Mahogany and Demi were under the similar evolutionary forces, which is also in accordance with their close genetic distance. High LD levels of Pastel and Black-NS might be due to the small number of founders initially brought to the CCFAR farm. In addition, the higher genetic differentiation of Pastel and Black-NS could lead to higher ALD for these color-types. The extension of *r*^2^ > 0.2 to genomic distances of 100 kb for Black-ON might be indicative of strong founder effects in this population. Generally, observation of long-range LD could be due to population admixture [73,74], random genetic drift [75], structural variations of genome [76,77], epistatic selection [78], hitchhiking effect of positively selected mutations [79] and demographic changes [80]. For instance, recurrent bottlenecks were suggested as the contributing force to long-range LD observed in the European human populations [81,82]. Furthermore, false appearance of LD could originate from some types of errors in the data or reference genome [82]. In the current study, the pattern of *Ne* as well as the abundance of shorter ROH (500 kb to 1 Mb) indicated that the domestication of Black-ON mink was likely initiated with a smaller number of founders or earlier than other groups. There is evidence that the LD curve is high and flat after the end of the population collapse and as population size increases at the end of the bottleneck, the right portion of the LD curve descends more quickly than the left portion [83], which is in accordance with the LD pattern observed for Black-ON in our study. Although it seems that association mapping may require the lower density of SNP markers in Black-ON population, the likelihood of false positive associations between markers and causal genes would increase due to the high level of LD. In other words, the hitchhiking effects of high LD haplotype-blocks could lead to an excess of candidate SNPs and less precise mapping of causative variants [83].

Results of this study confirmed no wide genetic distinction among the studied mink indicating that the samples might not be ideal to reveal the population structure. For instance, the existence of wild groups and samples from a wider geographical distribution could enrich the analysis of population history. In addition, the small sample size can result in biased and less accurate estimation of LD measures and the bias might vary with the distances between markers [84]. Although the *r*^2^ values in the current study were corrected for the sampling error, they might be subjected to bias and any comparison should be conservative. In fact, the LD estimations may vary depending on the sample size, MAF threshold, marker density and inter-marker distances applied in different studies. Despite all the restrictions, the main idea of this study was to provide the preliminary results for development of genomic evaluations in the mink industry. Given the fact that the mink industry has faced with economic issues in recent years, especially with the effect of SARS-Cov-2 on mink farms in Europe and North America, this industry will be required to increase the production efficiency. Genomic selection is suggested as an appropriate strategy to improve the economically important traits such as SARS-Cov-2 disease resistance and fur quality in mink [29]. The results of this study could provide the essential information to design the SNP panel for American mink and subsequently apply modern genomic strategies in the mink industry.

## 5. Conclusions

In this study, whole genome sequences were used to reveal the genetic structure and LD patterns of 100 American mink. The genetic analysis confirmed that there was no wide genetic differentiation among the studied individuals. However, three ancestral genetic compositions were recognized among the mink from two studied farms. Our analyses showed that the CCFAR is an admixed population whereas animals from Millbank Fur Farm were nearly pure. We observed a relationship between the admixture levels and LD extensions across the studied color-types where the three less admixed color-types (i.e., Pastel, Black-NS, and Black-ON) presented higher levels of LD and the highly admixed color-types (Mahogany and Demi) had lower LD levels. The extension of LD (*r*^2^) to genomic distances of 20 kb and 100 kb for CCFAR and Millbank Fur Farm suggested that 120,000 and 24,000 SNPs would require for applying genomic selection programs in these farms, respectively. These results indicated that accounting for admixture is critical to design the SNP panels and achieve the required accuracy in genomic studies of American mink.

## Figures and Tables

**Table 1:** Average minor allele frequency (MAF), observed heterozygosity, inbreeding rates based on excess of homozygosity (F_HOM_) and pedigree (F_PED_) for five color-types of American mink using whole genome sequence data.

Color-Types	Number of Animals	Average MAF	Observed Heterozygosity (%)	F_HOM_	F_PED_
Demi	32	0.214	31.12	−0.189	0.006
Pastel	10	0.195	29.42	−0.146	0.026
Mahogany	20	0.211	30.93	−0.184	0.009
Stardust	7	0.190	30.64	−0.153	0.016
Black-NS ^1^	16	0.197	31.32	−0.166	0.011
CCFAR ^2^	85	0.216	30.87	−0.171	0.010
Black-ON ^3^	15	0.192	28.03	−0.112	NA ^4^
Total	100	0.216	30.45	−0.166	-

^1^ Black color-type from Canadian Center for Fur Animal Research (CCFAR) at Dalhousie Faculty of Agriculture (Truro, NS, Canada); ^2^ All samples collected at Canadian Center for Fur Animal Research (CCFAR); ^3^ Black color-type from Millbank Fur Farm (Rockwood, ON, Canada); ^4^ Not available.

**Table 2:** The average (±SD) number of runs of homozygosity (ROH) and inbreeding rates based on ROH (F_ROH_) in different color-types of American mink.

Color-Types	Minimum Length of ROH					
	500 kb		1 Mb		2 Mb	
	Number	F_ROH_	Number	F_ROH_	Number	F_ROH_
Demi	61 ± 17	0.072 ± 0.022	19 ± 7	0.036 ± 0.013	3 ± 2	0.009 ± 0.006
Pastel	90 ± 21	0.110 ± 0.027	30 ± 8	0.057 ± 0.016	5 ± 2	0.016 ± 0.008
Mahogany	68 ± 24	0.078 ± 0.030	21 ± 10	0.039 ± 0.018	3 ± 1	0.009 ± 0.005
Stardust	85 ± 12	0.100 ± 0.014	29 ± 4	0.056 ± 0.009	3 ± 2	0.011 ± 0.007
Black-NS ^1^	82 ± 27	0.097 ± 0.034	26 ± 11	0.048 ± 0.021	3 ± 2	0.009 ± 0.007
CCFAR ^2^	72 ± 23	0.085 ± 0.029	23 ± 10	0.043 ± 0.018	3 ± 2	0.010 ± 0.006
Black-ON ^3^	120 ± 21	0.138 ± 0.031	39 ± 11	0.071 ± 0.019	4 ± 2	0.012 ± 0.007

^1^ Black color-type from Canadian Center for Fur Animal Research (CCFAR) at Dalhousie Faculty of Agriculture (Truro, NS, Canada); ^2^ All samples collected at Canadian Center for Fur Animal Research (CCFAR); ^3^ Black color-type from Millbank Fur Farm (Rockwood, ON, Canada).

**Table 3:** Estimation of Nei’s genetic distance (upper diagonal) and Weir and Cockerham’s Fst (lower diagonal) between different color-types of American mink.

Color-Types	Demi	Pastel	Mahogany	Stardust	Black-NS	Black-ON
Demi	0	0.025	0.013	0.036	0.029	0.030
Pastel	0.036	0	0.036	0.065	0.055	0.058
Mahogany	0.015	0.062	0	0.034	0.021	0.028
Stardust	0.055	0.124	0.046	0	0.039	0.044
Black-NS ^1^	0.057	0.114	0.033	0.066	0	0.037
Black-ON ^2^	0.059	0.122	0.048	0.077	0.076	0

^1^ Black color-type from Canadian Center for Fur Animal Research (CCFAR) at Dalhousie Faculty of Agriculture (Truro, NS, Canada); ^2^ Black color-type from Millbank Fur Farm (Rockwood, ON, Canada).

**Table 4:** Analysis of molecular variance (AMOVA) in different color-types of farmed mink.

Source of Variation	Degree of Freedom	Sum of Squares	Mean Squared Deviations	Variance Components	Percentage of Variation
Among farms	1	0.123	0.123	0.002	9
Among color-types	4	0.301	0.075	0.004 ***	18
Within color-types	94	1.560	0.016	0.016 ***	73
Total	99	1.984	0.020	0.022	100

*** *p* < 0.001.

**Table 5:** The average *r*^2^ ± SD between adjacent SNPs in five color-types of American mink.

Number of Scaffolds	Length (Mb)	Number of SNPs	Average *r*^2^ ± SD					
			Demi	Mahogany	Pastel	Black-NS ^1^	CCFAR ^2^	Black-ON ^3^
51	802.19	100,000	0.285 ± 0.346	0.280 ± 0.361	0.344 ± 0.411	0.343 ± 0.377	0.266 ± 0.334	0.366 ± 0.388

^1^ Black color-type from the Canadian Center for Fur Animal Research (CCFAR) at Dalhousie Faculty of Agriculture (Truro, NS, Canada); ^2^ All samples collected at the Canadian Center for Fur Animal Research (CCFAR); ^3^ Black color-type from Millbank Fur Farm (Rockwood, ON, Canada).

## Data Availability

The data presented in this study are available on request from the corresponding author. The data are not publicly available due to privacy.

## Supplementary Materials

The following are available online at https://www.mdpi.com/2073-4425/12/2/258/s1. Table S1: The average *r*^2^ ± SD between adjacent SNPs over different scaffolds in five color-types of American mink. Table S2: The average *r*^2^ ± SD over physical distances up to 1000 kb, pooled over all scaffolds, in five color-types of American mink.
