# Supplementary material for: Population Genomics of American Mink Using Whole Genome Sequencing Data
Source: Genes (Basel). 2021 Feb 11;12(2):258. doi: 10.3390/genes12020258 (PMC7916864; doi:10.3390/genes12020258)
Supplement: Supplementary file 1 [file genes-12-00258-s001.pdf]

Supplementary Materials

Table S1. Average r^2±SD between adjacent SNPs over different scaffolds in five color-types of American mink.

Scaffold number	Scaffold length (Mb)	Number of SNPs	Average r^2±SD					
			Demi	Pastel	Mahogany	Black-NS [1]	CCFAR [2]	Black-ON [3]
Scaffold 1	40.31	4,609	0.291±0.352	0.351±0.412	0.262±0.351	0.324±0.354	0.261±0.333	0.421±0.410
Scaffold 2	30.15	3,848	0.268±0.345	0.330±0.407	0.269±0.360	0.360±0.392	0.263±0.337	0.340±0.383
Scaffold 3	24.29	3,265	0.271±0.335	0.295±0.397	0.266±0.348	0.313±0.370	0.231±0.308	0.340±0.378
Scaffold 4	24.58	3,180	0.247±0.324	0.307±0.404	0.241±0.342	0.299±0.360	0.233±0.315	0.355±0.394
Scaffold 5	23.57	3,742	0.257±0.321	0.369±0.417	0.245±0.338	0.332±0.374	0.244±0.310	0.385±0.392
Scaffold 6	20.48	4,299	0.214±0.303	0.326±0.405	0.221±0.325	0.269±0.343	0.205±0.286	0.283±0.355
Scaffold 7	25.67	2,656	0.284±0.346	0.377±0.446	0.309±0.373	0.379±0.403	0.281±0.354	0.360±0.402
Scaffold 8	26.73	5,030	0.23±0.310	0.281±0.393	0.217±0.322	0.293±0.362	0.209±0.294	0.280±0.362
Scaffold 9	15.52	1,620	0.311±0.372	0.418±0.453	0.308±0.380	0.363±0.390	0.296±0.357	0.422±0.387
Scaffold 10	22.64	3,875	0.222±0.308	0.268±0.377	0.210±0.316	0.307±0.366	0.198±0.281	0.282±0.365
Scaffold 11	13.97	1,752	0.300±0.363	0.292±0.398	0.268±0.360	0.335±0.374	0.259±0.332	0.357±0.385
Scaffold 12	13.87	1,369	0.307±0.359	0.382±0.424	0.305±0.375	0.373±0.382	0.297±0.352	0.424±0.421
Scaffold 13	13.69	1,196	0.369±0.378	0.688±0.428	0.310±0.363	0.314±0.355	0.366±0.383	0.450±0.420
Scaffold 14	13.62	1,617	0.268±0.335	0.354±0.408	0.332±0.384	0.402±0.386	0.274±0.335	0.406±0.410
Scaffold 15	13.47	2,086	0.280±0.336	0.434±0.438	0.296±0.358	0.347±0.379	0.265±0.338	0.366±0.385
Scaffold 16	16.86	2,328	0.272±0.349	0.310±0.412	0.239±0.342	0.272±0.370	0.225±0.318	0.289±0.351
Scaffold 17	13.41	869	0.331±0.380	0.38±0.414	0.287±0.364	0.356±0.419	0.326±0.385	0.388±0.412
Scaffold 18	13.25	1,810	0.247±0.333	0.302±0.402	0.260±0.363	0.305±0.364	0.234±0.317	0.342±0.374
Scaffold 19	13.06	1,511	0.241±0.319	0.315±0.399	0.283±0.368	0.348±0.383	0.253±0.33	0.380±0.390
Scaffold 20	13.46	1,515	0.286±0.356	0.312±0.407	0.290±0.365	0.315±0.356	0.246±0.318	0.419±0.397
Scaffold 21	12.95	1,566	0.298±0.354	0.306±0.400	0.283±0.357	0.315±0.379	0.275±0.334	0.319±0.375
Scaffold 22	13.42	1,177	0.227±0.323	0.359±0.448	0.278±0.381	0.278±0.361	0.219±0.321	0.338±0.388
Scaffold 23	17.26	1,744	0.297±0.331	0.321±0.397	0.331±0.377	0.500±0.409	0.313±0.351	0.529±0.420
Scaffold 24	12.57	752	0.368±0.383	0.469±0.432	0.344±0.401	0.531±0.428	0.371±0.39	0.444±0.399
Scaffold 25	23.88	2,273	0.292±0.356	0.328±0.414	0.265±0.349	0.347±0.388	0.242±0.323	0.319±0.377
Scaffold 26	12.39	1,315	0.307±0.359	0.348±0.415	0.275±0.366	0.330±0.381	0.258±0.333	0.294±0.355
Scaffold 27	12.20	2,041	0.263±0.332	0.335±0.404	0.269±0.361	0.288±0.359	0.224±0.311	0.322±0.379
Scaffold 28	12.62	2,540	0.205±0.296	0.297±0.394	0.203±0.311	0.269±0.344	0.186±0.270	0.298±0.363
Scaffold 29	11.67	1,055	0.358±0.380	0.401±0.394	0.318±0.385	0.369±0.358	0.323±0.382	0.487±0.422
Scaffold 30	17.23	1,717	0.302±0.343	0.351±0.411	0.347±0.399	0.410±0.397	0.305±0.357	0.410±0.410
Scaffold 31	13.07	2,120	0.332±0.370	0.314±0.401	0.340±0.377	0.326±0.357	0.286±0.335	0.398±0.385
Scaffold 32	11.37	1,164	0.274±0.353	0.314±0.421	0.279±0.377	0.319±0.391	0.266±0.356	0.386±0.413
Scaffold 33	10.99	1,016	0.289±0.338	0.359±0.420	0.295±0.376	0.319±0.369	0.278±0.335	0.367±0.374
Scaffold 34	10.92	703	0.334±0.379	0.423±0.445	0.278±0.351	0.333±0.358	0.313±0.354	0.499±0.448
Scaffold 35	15.82	1,117	0.331±0.372	0.352±0.435	0.301±0.369	0.389±0.399	0.299±0.358	0.277±0.339
Scaffold 36	19.10	3,689	0.216±0.304	0.258±0.383	0.223±0.328	0.273±0.356	0.199±0.285	0.267±0.354
Scaffold 37	10.62	1,495	0.304±0.353	0.376±0.425	0.287±0.359	0.363±0.398	0.293±0.350	0.404±0.404
Scaffold 38	10.32	890	0.306±0.359	0.296±0.398	0.271±0.358	0.425±0.388	0.276±0.339	0.396±0.393
Scaffold 41	16.62	3,172	0.218±0.307	0.289±0.390	0.236±0.334	0.266±0.345	0.198±0.285	0.257±0.344
Scaffold 45	12.63	1,436	0.324±0.362	0.371±0.414	0.282±0.363	0.360±0.363	0.325±0.368	0.444±0.411
Scaffold 47	10.31	897	0.269±0.354	0.314±0.421	0.266±0.367	0.388±0.400	0.292±0.360	0.349±0.387
Scaffold 49	10.64	1,086	0.244±0.319	0.265±0.384	0.292±0.373	0.361±0.390	0.248±0.327	0.309±0.350
Scaffold 64	16.24	2,583	0.279±0.345	0.321±0.404	0.245±0.337	0.331±0.374	0.235±0.314	0.326±0.384
Scaffold 66	12.95	1,281	0.313±0.370	0.352±0.434	0.339±0.401	0.377±0.397	0.263±0.353	0.419±0.412
Scaffold 68	11.02	1,157	0.334±0.375	0.335±0.414	0.314±0.384	0.369±0.376	0.303±0.358	0.402±0.409
Scaffold 70	12.58	809	0.295±0.341	0.437±0.427	0.330±0.401	0.294±0.386	0.295±0.351	0.348±0.402

Scaffold 72	10.82	1,253	0.331±0.376	0.336±0.408	0.270±0.355	0.323±0.374	0.300±0.358	0.373±0.405
Scaffold 73	13.70	2,446	0.228±0.314	0.301±0.401	0.220±0.328	0.278±0.353	0.215±0.294	0.323±0.382
Scaffold 93	12.36	1,118	0.342±0.375	0.327±0.418	0.290±0.364	0.406±0.412	0.324±0.375	0.368±0.377
Scaffold 100	11.02	1,137	0.283±0.346	0.313±0.402	0.263±0.339	0.360±0.381	0.234±0.310	0.351±0.385
Scaffold 118	10.30	1,074	0.308±0.355	0.295±0.398	0.337±0.390	0.407±0.407	0.290±0.358	0.375±0.391
Overall	802.19	100,000	0.285±0.346	0.344±0.411	0.280±0.361	0.343±0.377	0.266±0.334	0.366±0.388

[1] Black color-type from the Canadian Center for Fur Animal Research (CCFAR) at Dalhousie Faculty of Agriculture (Truro, NS, Canada)

[2] All samples collected at the Canadian Center for Fur Animal Research (CCFAR)

[3] Black color-type from Millbank Fur Farm (Rockwood, ON, Canada).

Table S2. Average r^2±SD over physical distances up to 1000 kb, pooled over all scaffolds, in five color-types of American mink.

SNP pairs distance (kb)	Number of pairs	Average r^2±SD					
		Demi	Pastel	Mahogany	Black-NS [1]	CCFAR [2]	Black-ON [3]
0-10	161,027	0.300±0.348	0.361±0.412	0.296±0.365	0.360±0.378	0.285±0.338	0.375±0.389
10-20	147,651	0.214±0.293	0.286±0.384	0.213±0.315	0.281±0.344	0.201±0.277	0.290±0.356
20-30	143,869	0.180±0.266	0.251±0.367	0.186±0.293	0.254±0.327	0.171±0.249	0.258±0.340
30-40	141,881	0.164±0.251	0.233±0.356	0.167±0.276	0.234±0.319	0.153±0.231	0.239±0.329
40-50	140,009	0.154±0.243	0.225±0.351	0.155±0.267	0.224±0.311	0.145±0.222	0.227±0.322
50-60	140,577	0.147±0.235	0.219±0.348	0.147±0.261	0.215±0.305	0.136±0.213	0.221±0.318
60-70	139,520	0.141±0.229	0.215±0.345	0.142±0.253	0.208±0.300	0.130±0.203	0.217±0.315
70-80	137,748	0.135±0.223	0.212±0.343	0.138±0.249	0.200±0.297	0.126±0.199	0.210±0.309
80-90	137,607	0.134±0.221	0.200±0.335	0.135±0.248	0.198±0.294	0.122±0.194	0.205±0.308
90-100	136,311	0.129±0.217	0.199±0.334	0.127±0.239	0.194±0.292	0.119±0.191	0.202±0.304
100-200	1,426,073	0.117±0.204	0.191±0.330	0.121±0.231	0.187±0.286	0.109±0.179	0.187±0.295
200-300	1,345,259	0.106±0.190	0.181±0.321	0.109±0.218	0.174±0.276	0.100±0.167	0.170±0.282
300-400	1,320,384	0.099±0.181	0.177±0.319	0.103±0.211	0.170±0.272	0.093±0.158	0.159±0.273
400-500	1,301,079	0.095±0.177	0.170±0.314	0.099±0.206	0.165±0.268	0.089±0.153	0.147±0.264
500-600	1,287,134	0.092±0.173	0.169±0.313	0.096±0.203	0.159±0.263	0.086±0.150	0.142±0.259
600-700	1,275,665	0.089±0.170	0.169±0.313	0.093±0.201	0.157±0.260	0.083±0.146	0.137±0.255
700-800	1,270,504	0.086±0.166	0.162±0.307	0.090±0.197	0.153±0.257	0.080±0.142	0.130±0.248
800-900	1,257,777	0.084±0.163	0.161±0.306	0.086±0.192	0.151±0.256	0.078±0.139	0.126±0.244
900-1000	1,251,948	0.082±0.161	0.158±0.304	0.085±0.191	0.148±0.253	0.075±0.135	0.120±0.238

[1] Black color-type from the Canadian Center for Fur Animal Research (CCFAR) at Dalhousie Faculty of Agriculture (Truro, NS, Canada)

[2] All samples collected at the Canadian Center for Fur Animal Research (CCFAR)

[3] Black color-type from Millbank Fur Farm (Rockwood, ON, Canada).
